# A Comprehensive Prediction Model of Hydraulic Extended-Reach Limit Considering the Allowable Range of Drilling Fluid Flow Rate in Horizontal Drilling

**DOI:** 10.1038/s41598-017-03261-3

**Published:** 2017-06-08

**Authors:** Xin Li, Deli Gao, Xuyue Chen

**Affiliations:** 1 State Key Laboratory of Petroleum Resources and Engineering, China University of Petroleum, Beijing, 102249 P.R. China; 2 MOE Key Laboratory of Petroleum Engineering, China University of Petroleum, Beijing, 102249 P.R. China; 3 Department of Petroleum and Geosystems Engineering, The University of Texas at Austin, Austin, TX 78705 USA

## Abstract

Hydraulic extended-reach limit (HERL) model of horizontal extended-reach well (ERW) can predict the maximum measured depth (MMD) of the horizontal ERW. The HERL refers to the well’s MMD when drilling fluid cannot be normally circulated by drilling pump. Previous model analyzed the following two constraint conditions, drilling pump rated pressure and rated power. However, effects of the allowable range of drilling fluid flow rate (*Q*_*min*_ ≤ *Q* ≤ *Q*_*max*_) were not considered. In this study, three cases of HERL model are proposed according to the relationship between allowable range of drilling fluid flow rate and rated flow rate of drilling pump (*Q*_*r*_). A horizontal ERW is analyzed to predict its HERL, especially its horizontal-section limit (*L*_*h*_). Results show that when *Q*_*min*_ ≤ *Q*_*r*_ ≤ *Q*_*max*_ (Case I), *L*_*h*_ depends both on horizontal-section limit based on rated pump pressure (*L*_*h1*_) and horizontal-section limit based on rated pump power (*L*_*h2*_); when *Q*_*min*_ < *Q*_*max*_ < *Q*_*r*_ (Case II), *L*_*h*_ is exclusively controlled by *L*_*h1*_; while *L*_*h*_ is only determined by *L*_*h2*_ when *Q*_*r*_ < *Q*_*min*_ < *Q*_*max*_ (Case III). Furthermore, *L*_*h1*_ first increases and then decreases with the increase in drilling fluid flow rate, while *L*_*h2*_ keeps decreasing as the drilling fluid flow rate increases. The comprehensive model provides a more accurate prediction on HERL.

## Introduction

Longer horizontal-section length of horizontal extended-reach well (ERW) often means higher oil and gas output, and is also an economical choice for oil field development^1^. However, drilling engineers have no idea how far the horizontal ERW can extend. The hydraulic extended-reach limit (HERL) theory of the horizontal ERW can be used to predict the maximum measured depth (MMD) of horizontal ERW from the perspective of hydraulics, especially the bearing capacity of drilling pump.

Wang and Guo (2008) first proposed the concept and the computational model of HERL theory^2^. The HERL theory of the horizontal ERW can be summarized as follows. The horizontal ERW cannot extend without limitation. Drilling pump will stop circulating drilling fluid when the total pressure losses of circulation system exceed the rated pressure of drilling pump, which is a critical point. The measured depth of the horizontal ERW at the critical point is defined as the HERL of the horizontal ERW. In other words, the HERL of the horizontal ERW refers to the well’s MMD when the drilling fluid cannot be normally circulated by drilling pump. The HERL is mainly related to the total pressure losses of circulation system and the rated pressure of the drilling pump. Later in 2009, Guo and Wang (2009) applied the HERL theory to the Liuhua field in the South China Sea^3^. They analyzed the ERW’s HERL based on the established HERL model. Gao *et al*. (2009) also introduced and analyzed the concept and influence factors in the HERL model for horizontal ERW^4^. Sun (2013) further developed the HERL model, he regarded the rated power of drilling pump as a new constraint condition^5^. The HERL model based on new constraint condition is also introduced. However, these studies only consider the effects of rated pressure of drilling pump and rated power of drilling pump on the HERL model; the allowable range of drilling fluid flow rate, an important hydraulic parameter range, was not taken into consideration.

Each drilling pump has a maximum output power, known as the rated power of drilling pump *P*_*r*_. Meanwhile, each drilling pump also possesses several cylinders with different diameters, and every cylinder has a certain allowable pressure, which is called the rated pressure of drilling pump *p*_*r*_. The drilling fluid flow rate *Q* under the conditions of *P*_*r*_ and *p*_*r*_ is called the rated flow rate of drilling pump *Q*_*r*_. In general, *P*_*r*_, *p*_*r*_ and *Q*_*r*_ have the following relationship.

1 $${P}_{r}={p}_{r}{Q}_{r}$$

When $Q\le {Q}_{r}$, the pump pressure is restricted by the allowable pressure of cylinder, the maximum pump pressure can only reach the rated pump pressure of drilling pump *p*_*r*_. Then the pump power keeps increases with the increase in drilling fluid flow rate *Q* until $Q={Q}_{r}$, namely the rated power of drilling pump *P*_*r*_ is reached, and the drilling fluid flow rate *Q* at this time is the rated flow rate of drilling pump *Q*_*r*_. In brief, *p*_*r*_ is the major constraint condition when $Q\le {Q}_{r}$. In contrast, the pump power is maintained at *P*_*r*_ when $Q > {Q}_{r}$, the pump pressure keeps decreasing as drilling fluid flow rate *Q* increase. In other words, *P*_*r*_ becomes the main constraint condition when *Q* > *Q*_*r*_^6^.

As mentioned above, the rated pump pressure of drilling pump *p*_*r*_ and the rated power of drilling pump *P*_*r*_ as two constraint conditions of HERL model for horizontal ERW are provided under the conditions of *Q* ≤ *Q*_*r*_ and *Q* > *Q*_*r*_ respectively. However, the effects of allowable range of drilling fluid flow rate on the HERL model are not considered. During the drilling process, the drilling fluid flow rate *Q* has a theoretical range, namely the allowable range of drilling fluid flow rate. Specifically, too small *Q* cannot meet the needs of hole cleaning; however, if *Q* is too large, the bearing capacity of the drilled formation may be threatened. The allowable range of drilling fluid flow rate is expressed in Eq. (2).

2 $${Q}_{{\rm{\min }}}\le Q\le {Q}_{{\rm{\max }}}$$

where *Q*_min_ is the lower limit of drilling fluid flow rate, L/s; *Q*_max_ is the upper limit of drilling fluid flow rate, namely the upper limit considering the bearing capacity of drilled formation, L/s.

The main purpose of this paper is to establish a more comprehensive and accurate model of HERL for horizontal ERW according to the relationship between the above allowable range of drilling fluid flow rate and the rated flow rate of drilling pump *Q*_*r*_. Moreover, the bearing capacity of existing hydraulic equipment can also be evaluated based on the established HERL model, avoiding the situation that the designed horizontal-section length exceeds the limit extension ability provided by the available drilling pump.

## Results

### HERL model

For a horizontal ERW, the lengths of vertical section and deviated sections can be obtained by an inclinometer before drilling into the horizontal section. Therefore, we mainly analyze the well’s horizontal-section limit *L*_*h*_, which can be expressed in Eq. (3).

3 $$\{\begin{array}{c}{L}_{h}={L}_{h1}=\frac{{p}_{r}-{\rm{\Delta }}{p}_{g}-{\rm{\Delta }}{p}_{b}-({\rm{\Delta }}{p}_{stv}+{\rm{\Delta }}{p}_{std})-({\rm{\Delta }}{p}_{av}+{\rm{\Delta }}{p}_{ads}+{\rm{\Delta }}{p}_{adl})}{{(\frac{{\rm{\Delta }}p}{{\rm{\Delta }}L})}_{sth}+{(\frac{{\rm{\Delta }}p}{{\rm{\Delta }}L})}_{ah}},(Q\le {Q}_{r})\\ {L}_{h}={L}_{h2}=\frac{\frac{{P}_{r}}{Q}-{\rm{\Delta }}{p}_{g}-{\rm{\Delta }}{p}_{b}-({\rm{\Delta }}{p}_{stv}+{\rm{\Delta }}{p}_{std})-({\rm{\Delta }}{p}_{av}+{\rm{\Delta }}{p}_{ads}+{\rm{\Delta }}{p}_{adl})}{{(\frac{{\rm{\Delta }}p}{{\rm{\Delta }}L})}_{sth}+{(\frac{{\rm{\Delta }}p}{{\rm{\Delta }}L})}_{ah}},(Q > {Q}_{r})\\ {Q}_{{\rm{\min }}}\le Q\le {Q}_{{\rm{\max }}}\end{array}$$

where *L*_*h*_ is the horizontal-section limit, m; *L*_*h*1_ is the horizontal-section limit based on rated pump pressure, m; ${L}_{h2}$ is the horizontal-section limit based on rated pump power, m; *p*_*r*_ is the rated pressure of drilling pump, MPa; *P*_*r*_ is the rated power of drilling pump, kW; ${\rm{\Delta }}{p}_{g}$ is surface pipeline pressure drop, MPa; ${\rm{\Delta }}{p}_{b}$ is bit pressure drop, MPa; ${\rm{\Delta }}{p}_{stv}$ is the drill string pressure losses of vertical section, MPa; ${\rm{\Delta }}{p}_{std}$ is the drill string pressure losses of deviated sections, MPa; ${\rm{\Delta }}{p}_{av}$ is the annular pressure losses of vertical section, MPa; ${\rm{\Delta }}{p}_{ads}$ is annular pressure losses of small-inclination section, MPa; ${\rm{\Delta }}{p}_{adl}$ is annular pressure losses of large-inclination section, MPa; ${(\frac{{\rm{\Delta }}p}{{\rm{\Delta }}L})}_{sth}$ is drill string pressure loss gradients in horizontal section, MPa/m; ${(\frac{{\rm{\Delta }}p}{{\rm{\Delta }}L})}_{ah}$ is annular pressure loss gradients in horizontal section, MPa/m.

According to the relationship between the allowable range of drilling fluid flow rate ${Q}_{{\rm{\min }}}\le Q\le {Q}_{{\rm{\max }}}$ and the rated flow rate of drilling pump *Q*_*r*_, the Eq. (3) can be divided into the following three Cases, including ${Q}_{{\rm{\min }}}\le {Q}_{r}\le {Q}_{{\rm{\max }}}$, ${Q}_{\min } < {Q}_{\max } < {Q}_{r}$ and ${Q}_{r} < {Q}_{\min } < {Q}_{\max }$. They are expressed in Eqs (4)–(6).

Case I: $${Q}_{{\rm{\min }}}\le {Q}_{r}\le {Q}_{{\rm{\max }}}$$;

4 $${L}_{h}=\{\begin{array}{c}{L}_{h1}=\frac{{p}_{r}-{\rm{\Delta }}{p}_{g}-{\rm{\Delta }}{p}_{b}-({\rm{\Delta }}{p}_{stv}+{\rm{\Delta }}{p}_{std})-({\rm{\Delta }}{p}_{av}+{\rm{\Delta }}{p}_{ads}+{\rm{\Delta }}{p}_{adl})}{{(\frac{{\rm{\Delta }}p}{{\rm{\Delta }}L})}_{sth}+{(\frac{{\rm{\Delta }}p}{{\rm{\Delta }}L})}_{ah}},({Q}_{{\rm{\min }}}\le Q\le {Q}_{r})\\ {L}_{h2}=\frac{\frac{{P}_{r}}{Q}-{\rm{\Delta }}{p}_{g}-{\rm{\Delta }}{p}_{b}-({\rm{\Delta }}{p}_{stv}+{\rm{\Delta }}{p}_{std})-({\rm{\Delta }}{p}_{av}+{\rm{\Delta }}{p}_{ads}+{\rm{\Delta }}{p}_{adl})}{{(\frac{\Delta p}{{\rm{\Delta }}L})}_{sth}+{(\frac{{\rm{\Delta }}p}{{\rm{\Delta }}L})}_{ah}},({Q}_{r} < Q\le {Q}_{{\rm{\max }}})\end{array}$$

where *Q*_*r*_ is rated flow rate of drilling pump, L/s.

Case II: $${Q}_{\min } < {Q}_{\max } < {Q}_{r}$$;

5 $$\begin{array}{cc}{L}_{h}={L}_{h1}=\frac{{p}_{r}-{\rm{\Delta }}{p}_{g}-{\rm{\Delta }}{p}_{b}-({\rm{\Delta }}{p}_{stv}+{\rm{\Delta }}{p}_{std})-({\rm{\Delta }}{p}_{av}+{\rm{\Delta }}{p}_{ads}+{\rm{\Delta }}{p}_{adl})}{{(\frac{\Delta p}{\Delta L})}_{sth}+{(\frac{\Delta p}{\Delta L})}_{ah}}, & \,({Q}_{{\rm{\min }}}\le Q\le {Q}_{{\rm{\max }}} < {Q}_{r})\end{array}$$

Case III: $${Q}_{r} < {Q}_{\min } < {Q}_{\max }$$;

6 $${L}_{h}={L}_{h2}=\frac{\frac{{P}_{r}}{Q}-{\rm{\Delta }}{p}_{g}-{\rm{\Delta }}{p}_{b}-({\rm{\Delta }}{p}_{stv}+{\rm{\Delta }}{p}_{std})-({\rm{\Delta }}{p}_{av}+{\rm{\Delta }}{p}_{ads}+{\rm{\Delta }}{p}_{adl})}{{(\frac{{\rm{\Delta }}p}{{\rm{\Delta }}L})}_{sth}+{(\frac{{\rm{\Delta }}p}{{\rm{\Delta }}L})}_{ah}},({Q}_{r} < {Q}_{\min }\le Q\le {Q}_{\max })$$

### Application example

**Figure 1:**
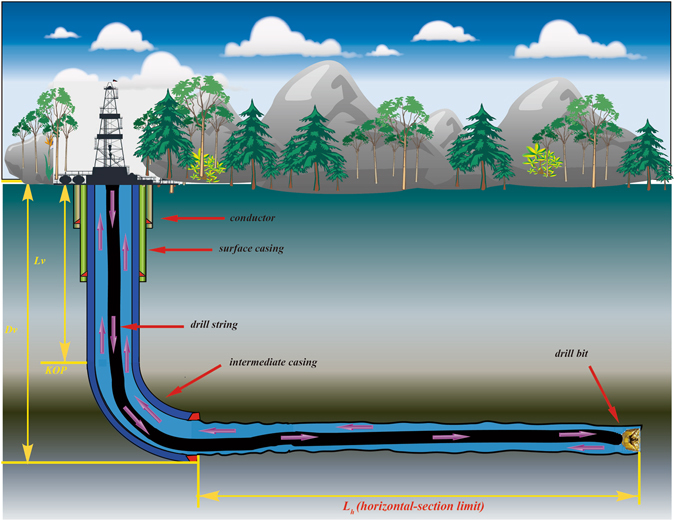
Schematic overview of the horizontal extended-reach well.

For a horizontal ERW, the established HERL model is used to predict the well’s HERL, especially the horizontal-section limit. The specific data of this well is listed in Tables 1 and 2 ^7^, and schematic overview of the horizontal ERW is illustrated in Fig. 1.

**Table 1:** Design table of casing program.

Casing program	Bit size/mm	Casing outer diameter/mm	Casing depth/m
Conductor	558.8	476.3	30
Surface casing	444.5	339.7	700
Intermediate casing	311.2	244.5	2407
Open hole	215.9	—	—

**Table 2:** List of input data for modeling.

Variables	Value	Unit
Inclination at KOP	0	°
*L*_*v*_	1956.3	m
Build rate	20.55	°/100 m
Inclination at target base	90	°
True vertical depth *D*_*v*_	2241	m
Horizontal displacement before the horizontal section	280	m
Drilling fluid density *ρ*_*m*_	1.35	g/cm^3^
Cuttings density *ρ*_*s*_	2.5	g/cm^3^
Flow behavior index *n*	0.7365	—
Consistency coefficient *K*	0.7565	Pa·s^n^
Fracture pressure equivalent density *ρ*_*f*_	1.91	g/cm^3^
Designed horizontal-section length *L*_*h0*_	1500	m
Drill pipe outer diameter *D*_*i*_	139.7	mm
Drill pipe rotation speed *N*	40	rpm
Rate of penetration *ROP*	10	m/h
Rated pump pressure *p*_*r*_	39	MPa
Rated pump power *P*_*r*_	1323	kW

First of all, the authors assume that the fracture pressure in the horizontal section is identical, otherwise inconsistent comparison conditions will occur when the parameters sensitivity analysis is carried out. Meanwhile, the bearing capacity of drilled formation and the needs of hole cleaning should be considered to determine the allowable range of drilling fluid flow rate.

**Figure 2:**
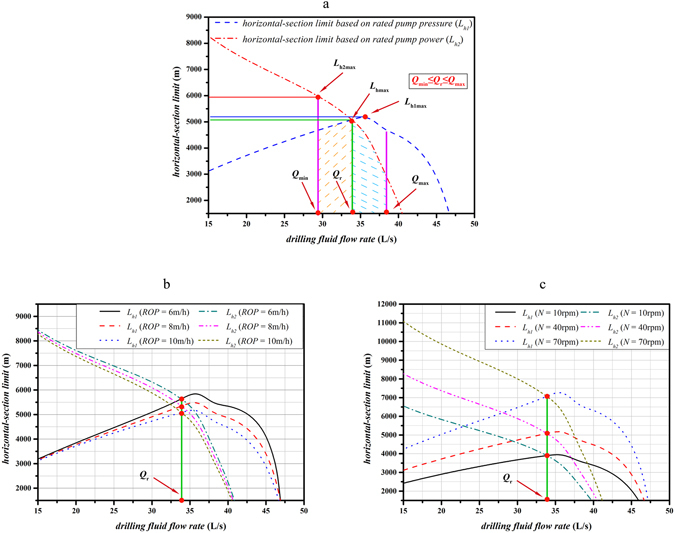
Effects of drilling fluid flow rate on *L*_*h1*_ and *L*_*h2*_ & the schematic overview of the situation *Q*_*min*_ ≤ *Q*_*r*_ ≤ *Q*_*max*_ (Case I). (**a**) Effects of drilling fluid flow rate on *L*_*h1*_ and *L*_*h2*_; (**b**) Effects of different ROPs on *L*_*h1*_ and *L*_*h2*_; (**c**) Effects of different drill pipe rotation speeds on *L*_*h1*_ and *L*_*h2*_.

The specific calculation results show that the lower limit based on the needs of hole cleaning ${Q}_{hc}$ is 29.6 L/s, the lower limit considering the bearing capacity of drilled formation ${Q}_{\min -df}$ is 27.1 L/s, and the upper limit of drilling fluid flow rate ${Q}_{\max }$ is 39 L/s. Therefore, the allowable range of drilling fluid flow rate ranges from 29.6 L/s to 38.5 L/s. Moreover, according to the conditions given in Tables 1 and 2, the rated pressure of drilling pump *p*_*r*_ is 39 MPa, the rated power of drilling pump *P*_*r*_ is 1323 kW, so the rated flow rate of drilling pump *Q*_*r*_ is 34 L/s. Depending on the relationship between allowable range of drilling fluid flow rate and the rated flow rate of drilling pump *Q*_*r*_, the HERL model belongs to Case I, which can be determined by Eq. (4). Effects of drilling fluid flow rate on the horizontal-section limit based on rated pump pressure *L*_*h*1_ and the horizontal-section limit based on rated pump power *L*_*h*2_ are shown in Fig. 2a, which is also the schematic overview of the situation of ${Q}_{\min }\le {Q}_{r}\le {Q}_{\max }$ (Case I).

As shown in the Fig. 2a, the horizontal-section limit based on rated pump pressure ${L}_{h1}$ first increases and then decreases with increase in drilling fluid flow rate; meanwhile, the horizontal-section limit based on rated pump power ${L}_{h2}$ keep decreasing as drilling fluid drilling fluid flow rate increases when ${Q}_{\min }\le {Q}_{r}\le {Q}_{\max }$. The abscissa value of the intersection between these two curves is the rated flow rate of drilling pump *Q*_*r*_ (34 L/s). The HERL, especially the horizontal-section limit is mainly dependent on ${L}_{h1}$ when *Q* ranges from ${Q}_{\min }$ to *Q*_*r*_ (${Q}_{\min }\le Q\le {Q}_{r}$), which is indicated by the yellow dotted area in the Fig. 2a. However, the HERL especially the horizontal-section limit mainly depends on ${L}_{h2}$ if *Q* ranges from *Q*_*r*_ to ${Q}_{\max }$ (${Q}_{r} < Q\le {Q}_{\max }$), which is indicated by the blue dotted area in the Fig. 2a. Furthermore, both ${L}_{{h}{\rm{1max}}}$ (the maximum horizontal-section limit based on rated pump pressure) and ${L}_{h2\max }$ (the maximum horizontal-section limit based on rated pump power) are larger than ${L}_{{h}{\rm{\max }}}$ (the maximum horizontal-section limit). ${L}_{{h}{\rm{\max }}}$ can be obtained at ${Q}_{r}$ (34 L/s). Specifically, ${L}_{h{\rm{1max}}}$ is 5270 m, ${L}_{h{\rm{2max}}}$ is 5955 m, while ${L}_{{h}{\rm{\max }}}$ is 5068 m. Considering the lengths of vertical section and deviated sections, each drilling fluid flow rate corresponds to a well’s HERL, and the maximum HERL of the horizontal ERW is 7463 m, which can also be obtained at *Q*_*r*_ (34 L/s).

## Discussion

In order to analyze the effects of different parameters on the HERL especially the horizontal-section limit of horizontal ERW, parameters sensitivity analysis is discussed. Furthermore, results simulated by the established model are also compared with the results of the previous model that did not consider the allowable range of drilling fluid flow rate.

### Effects of rate of penetration on HERL

Rate of penetration (ROP) is of significance to the economic benefits in drilling engineering. First, allowable ranges of drilling fluid flow rate under different ROPs (6 m/h, 8 m/h, 10 m/h) are listed in Table 3.

**Table 3:** Allowable ranges of drilling fluid flow rate under different parameters.

Variables	Results	Lower limit of drilling fluid flow rate *Q*_*min*_ (L/s)			Upper limit of drilling fluid flow rate *Q*_*max*_ (L/s)	Rated flow rate *Q*_*r*_ (L/s)	Situation	Case
		*Q*_*min-df*_	*Q*_*hc*_	*Q*_*min*_				
*ROP* (m/h)	6	26.1	28.6	28.6	40.0	34	*Q*_*min*_ ≤ *Q*_*r*_ ≤ *Q*_*max*_	I
	8	26.4	29.1	29.1	39.2	34	*Q*_*min*_ ≤ *Q*_*r*_ ≤ *Q*_*max*_	I
	10	27.1	29.6	29.6	38.4	34	*Q*_*min*_ ≤ *Q*_*r*_ ≤ *Q*_*max*_	I
*N* (rpm)	10	31.5	31.1	31.5	40.9	34	*Q*_*min*_ ≤ *Q*_*r*_ ≤ *Q*_*max*_	I
	40	26.9	29.6	29.6	41.3	34	*Q*_*min*_ ≤ *Q*_*r*_ ≤ *Q*_*max*_	I
	70	21.1	28.2	28.2	41.8	34	*Q*_*min*_ ≤ *Q*_*r*_ ≤ *Q*_*max*_	I
*p*_*r*_ (MPa)	39	27.1	29.6	29.6	38.5	34	*Q*_*min*_ ≤ *Q*_*r*_ ≤ *Q*_*max*_	I
	34	27.1	29.6	29.6	38.5	39	*Q*_*min*_ < *Q*_*max*_ < *Q*_*r*_	II
	31	27.1	29.6	29.6	38.5	43	*Q*_*min*_ < *Q*_*max*_ < *Q*_*r*_	II
*P*_*r*_ (kW)	1323	27.1	29.6	29.6	38.5	34	*Q*_*min*_ ≤ *Q*_*r*_ ≤ *Q*_*max*_	I
	1049	27.1	29.6	29.6	38.5	26.9	*Q*_*r*_ < *Q*_*min*_ < *Q*_*max*_	III
	726	27.1	29.6	29.6	38.5	18.6	*Q*_*r*_ < *Q*_*min*_ < *Q*_*max*_	III
*L*_*h0*_ (m)	1500	27.1	29.6	29.6	38.5	34	*Q*_*min*_ ≤ *Q*_*r*_ ≤ *Q*_*max*_	I
	3000	34.9	29.6	34.9	37.6	34	*Q*_*r*_ < *Q*_*min*_ < *Q*_*max*_	III
	6000	—	29.6	—	—	34	—	—

As shown in Table 3, the lower limit of drilling fluid flow rate ${Q}_{\min }$ gradually increases and the upper limit of drilling fluid flow rate ${Q}_{\max }$ keeps decreasing as ROP increases. In other words, the window of drilling fluid flow rate becomes narrower. The effects of different ROPs on ${L}_{h1}$ and ${L}_{h2}$ are shown in the Fig. 2b.

As shown in the Fig. 2b, ${L}_{h1}$ first increases and subsequently decreases with the increase in drilling fluid flow rate, whereas ${L}_{h2}$ keep decreases with the increase in drilling fluid flow rate. Moreover, both ${L}_{h1}$ and ${L}_{h2}$ have a negative correlation with ROP under the condition of identical drilling fluid flow rate, since the annular cuttings and the annular pressure losses increase with the increase in ROP. In addition, ROP has no effects on the rated flow rate of drilling pump *Q*_*r*_, and *Q*_*r*_ = 34 L/s. The horizontal-section limit here can be determined by the Eq. (4) (Case I), and the maximum horizontal-section limit ${L}_{{h}{\rm{\max }}}$ can be achieved at *Q*_*r*_ with different ROPs.

### Effects of drill pipe rotation speed on HERL

Results of allowable ranges of drilling fluid flow rate under different drill pipe rotation speeds (10 rpm, 40 rpm, 70 rpm) are listed in Table 3.

Table 3 shows that the lower limit of drilling fluid flow rate ${Q}_{\min }$ decreases and the upper limit of drilling fluid flow rate ${Q}_{\max }$ increases with the increase in drill pipe rotation speed *N*. In other words, the window of drilling fluid flow rate becomes wider. The effects of different drill pipe rotation speeds on ${L}_{h1}$ and ${L}_{h2}$ are shown in Fig. 2c.

Similarly, the Fig. 2c shows that ${L}_{h1}$ begins to decrease as drilling fluid flow rate increases after ${L}_{h1}$ reached its upper limit, whereas ${L}_{h2}$ has a consistent negative correlation with drilling fluid flow rate. Moreover, the rotation of drill pipe is conductive to the efficiency of hole cleaning, as a result of which both ${L}_{h1}$ and ${L}_{h2}$ increase with the increase in drill pipe rotation speed *N* under the condition of identical drilling fluid flow rate. Furthermore, drill pipe rotation speed also has no effects on rated flow rate of drilling pump *Q*_*r*_. The horizontal-section limit here can also be determined by the Eq. (4) (Case I) and the maximum horizontal-section limit ${L}_{h\max }$ can be achieved at *Q*_*r*_ = 34 L/s with different drill pipe rotation speeds.

### Effect of rated pressure of drilling pump on HERL

The rated pressure of drilling pump ${p}_{r}$, an important parameter of the HERL model, has great effects on the HERL of horizontal ERW especially the horizontal-section limit ${L}_{h}$. First of all, allowable ranges of drilling fluid flow rate under different rated pump pressures are calculated and listed in Table 3.

**Figure 3:**
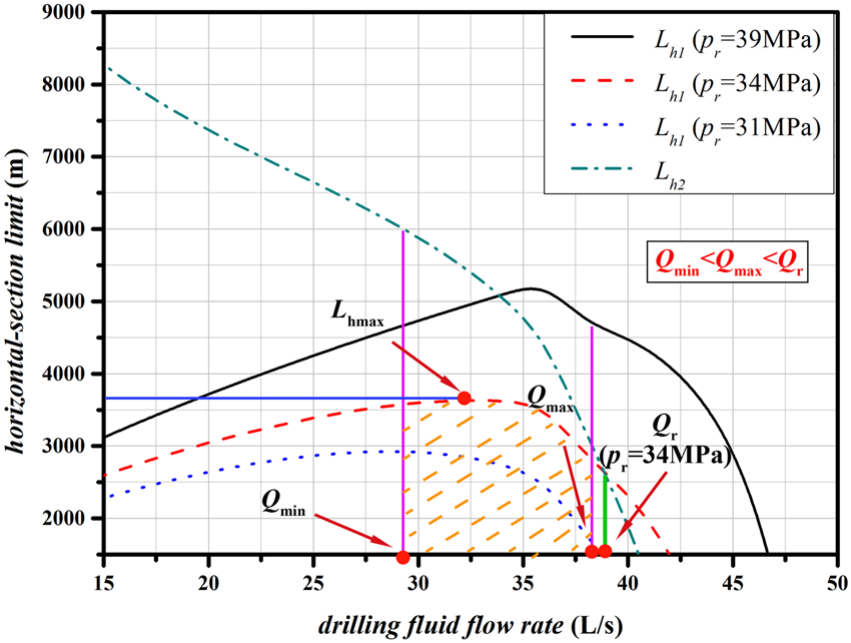
Schematic overview of the situation *Q*_*min*_ < *Q*_*max*_ < *Q*_*r*_ (Case II) and effects of different rated pump pressures on *L*_*h1*_ and *L*_*h2*_.

Table 3 shows that *p*_*r*_ has no effects on hole cleaning and the bearing capacity of the drilled formation. Moreover, different *p*_*r*_ correspond to different *Q*_*r*_. Specifically, *Q*_*r*_ = 34 L/s when *p*_*r*_ = 39 MPa, *Q*_*r*_ = 39 L/s when *p*_*r*_ = 34 MPa and *Q*_*r*_ = 43 L/s when *p*_*r*_ = 31 MPa. The situation of *p*_*r*_ = 39 MPa is exactly the same as that in Fig. 2a. The situation of *p*_*r*_ = 34 MPa is focused in this part, the horizontal-section limit ${L}_{h}$ can be calculated by Eq. (5) since ${Q}_{\min } < {Q}_{\max } < {Q}_{r}$. The effects of different rated pump pressures on ${L}_{h1}$ and ${L}_{h2}$ are illustrated in Fig. 3, which is also the schematic overview of situation ${Q}_{\min } < {Q}_{\max } < {Q}_{r}$ (Case II).

Figure 3 shows that ${L}_{h1}$ first increases, but later decreases with the increase in drilling fluid flow rate, while ${L}_{h2}$ keep decreasing as drilling fluid flow rate increase. Moreover, the higher *p*_*r*_ corresponds to the greater ${L}_{h1}$ when drilling fluid flow rates are the same. However, *p*_*r*_ has no effects on ${L}_{h2}$.

According to the Eq. (5), the horizontal-section limit totally depends on ${L}_{h1}$ when ${Q}_{\min } < {Q}_{\max } < {Q}_{r}$ (Case II), which is indicated by the yellow dotted area in the Fig. 3. Therefore, the maximum horizontal-section limit ${L}_{h\max }$ can be obtained at the drilling fluid flow rate *Q* when ${L}_{h1\max }$ is achieved rather than at the rated flow rate of drilling pump *Q*_*r*_.

### Effect of rated power of drilling pump on HERL

Firstly, allowable ranges of drilling fluid flow rate under different rated pump powers *P*_*r*_ are calculated. Which situation the HERL belongs to and which kind of HERL model needs to be adopted can be determined according to the relationships between these allowable ranges of drilling fluid flow rate and the rated flow rate of the drilling pump *Q*_*r*_.

**Figure 4:**
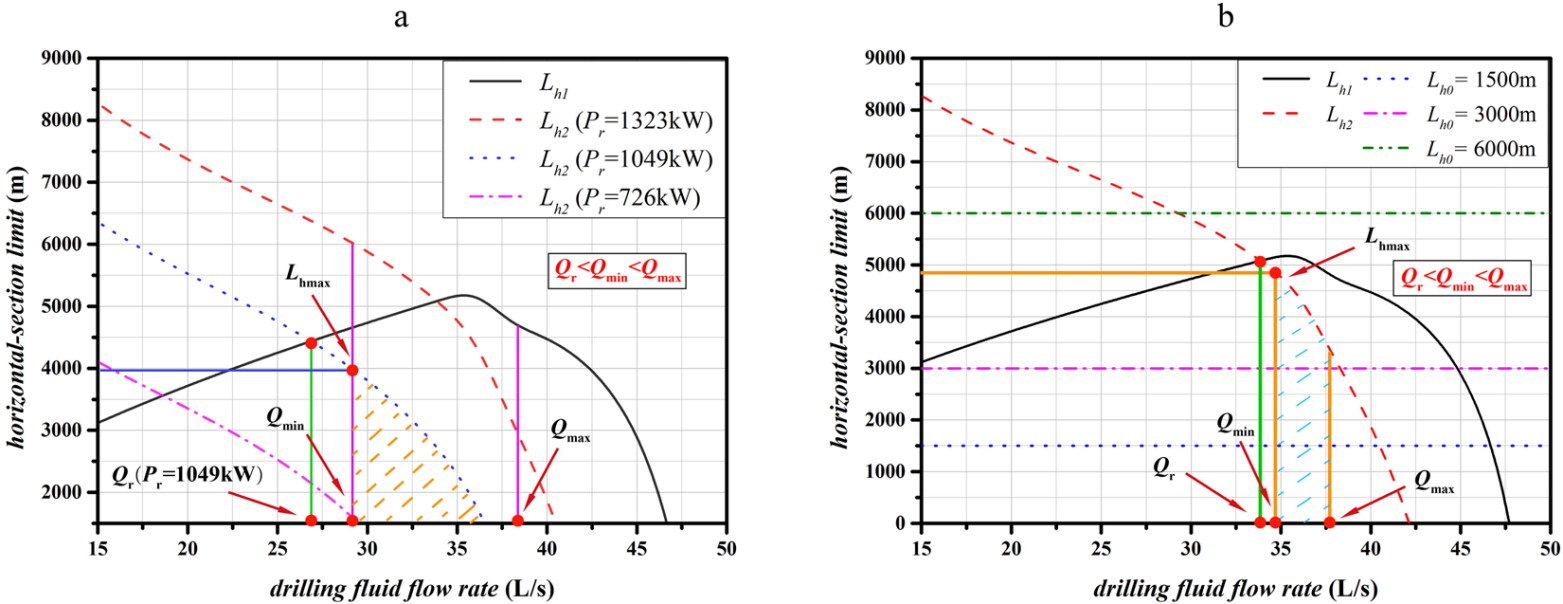
Schematic overview of the situation *Q*_*r*_ < *Q*_*min*_ < *Q*_*max*_ (Case III). (**a**) Effects of different rated pump powers on *L*_*h1*_ and *L*_*h2*_; (**b**) Effects of different designed horizontal-section lengths on *L*_*h1*_ and *L*_*h2*_.

Table 3 shows that rated pressure of drilling pump *P*_*r*_ has no effects on hole cleaning and the bearing capacity of drilled formation. The rated flow rate of drilling pump ${Q}_{r}=34L/{\rm{s}}$ when ${P}_{r}=1323{\rm{kW}}$, ${Q}_{r}=26.9L/{\rm{s}}$ when ${P}_{r}=1049{\rm{kW}}$ and ${Q}_{r}=18.6L/{\rm{s}}$ when ${P}_{r}=726{\rm{kW}}$. Their HERL Cases are listed in Table 3. The situation of ${P}_{r}=1049{\rm{kW}}$ is focused in this part, which belongs to the situation of ${Q}_{r} < {Q}_{\min } < {Q}_{\max }$ (Case III), and the horizontal-section limit can be determined by Eq. (6). The effects of rated pressure of drilling pump *P*_*r*_ on ${L}_{h1}$ and ${L}_{h2}$ are shown in Fig. 4a.

Similarly, the Fig. 4a shows that ${L}_{h1}$ first increases and subsequently decreases with the increase in drilling fluid flow rate, whereas ${L}_{h2}$ has a consistent negative correlation with drilling fluid flow rate. Moreover, the higher rated pressure of drilling pump *P*_*r*_ means the greater ${L}_{h2}$ with identical drilling fluid flow rate. However, *P*_*r*_ has no effects on ${L}_{h1}$.

According to the Eq. (6), the horizontal-section limit ${L}_{h}$ depends entirely on ${L}_{h2}$ when ${Q}_{r} < {Q}_{\min } < {Q}_{\max }$, which is indicated by the yellow dotted area in the Fig. 4a. Therefore, the maximum horizontal-section limit ${L}_{h\max }$ can be obtained at the drilling fluid flow rate *Q* when ${L}_{h2\max }$ is achieved rather than the rated flow rate of drilling pump *Q*_*r*_. The Fig. 4a shows that the horizontal-section limit ${L}_{h}$ at *Q*_*r*_ is larger than ${L}_{h\max }$ when ${P}_{r}=1049{\rm{kW}}$. Therefore, if the allowable range of drilling fluid flow rate is not considered, and taking the horizontal-section limit ${L}_{h}$ at *Q*_*r*_ as the ${L}_{h\max }$ when ${P}_{r}=1049{\rm{kW}}$, it will result in the designed horizontal-section length ${L}_{h0}$ being larger than the horizontal-section limit ${L}_{h}$ that can be achieved, and causing safety hazards.

### Effects of designed horizontal-section length on HERL

In general, different designed horizontal-section lengths ${L}_{h0}$ have great effects on drilling operations. In the part of application example, the designed horizontal-section length ${L}_{h0}$ is 1500 m. Allowable ranges of drilling fluid flow rate under different designed horizontal-section lengths (1500 m, 3000 m, 6000 m) are listed in Table 3.

Table 3 shows that the window of drilling fluid flow rate becomes narrower when the designed horizontal-section length ${L}_{h0}$ increases. Effects of different designed horizontal-section lengths ${L}_{h0}$ on the horizontal-section limit based on rated pump pressure ${L}_{h1}$ and the horizontal-section limit based on rated pump power ${L}_{h2}$ are illustrated in Fig. 4b.

As shown in the Fig. 4b, after the increase of ${L}_{h1}$ in the first stage, it begins to decreases as drilling fluid flow rate increase, while ${L}_{h2}$ keep decreasing as drilling fluid flow rate increases. When ${L}_{h0}=3000\,{\rm{m}}$, the lower limit of drilling fluid flow rate ${Q}_{\min }$ is 34.9 L/s, and the rated flow rate of drilling pump *Q*_*r*_ is 34 L/s, which belongs to the situation of ${Q}_{r} < {Q}_{\min } < {Q}_{\max }$ (Case III). The horizontal-section limit ${L}_{h}$ here can be determined by Eq. (6), and it is mainly dependent on ${L}_{h2}$ (seen from the Fig. 4b). The maximum horizontal-section limit ${L}_{h\max }$ can be achieved at the lower limit of drilling fluid flow rate ${Q}_{\min }$, rather than the rated flow rate of drilling pump *Q*_*r*_.

As shown in Table 3, the situation of ${L}_{h0}=1500\,\,{\rm{m}}$ is analyzed in the part of application example. However, drilling fluid flow rate window closed when ${L}_{h0}=6000\,{\rm{m}}$. At this point, the bottom hole pressure with any drilling fluid flow rate will exceed the bearing capacity of drilled formation. Moreover, there is no intersection between the curve of ${L}_{h1}$ and the straight line of ${L}_{h0}=6000\,{\rm{m}}$, indicating that drilling pump can no longer work at the rated pressure state of 39 MPa regardless of any drilling fluid flow rate.

### Comparison of the established model and the previous model

In the previous model, since the allowable range of drilling fluid flow rate is not considered, the drilling fluid flow rate *Q* can be considered in the range of zero to infinity, including the rated flow rate of drilling pump ${Q}_{r}$. The maximum horizontal-section limit ${L}_{{h}{\rm{\max }}}$ is achieved at *Q*_*r*_ when the allowable range of drilling fluid flow rate is not considered. In other words, the previous model can be taken as the Case I in the established model in this study. The previous model has been applied in the South China Sea. If the rated power of drilling pump *P*_*r*_ is 1049 kW and other conditions remain the same as those in the application example, ${L}_{h\max }$ is 4438 m based on the previous model, which can be obtained at *Q*_*r*_ of 26.9 L/s. But in fact, the lower limit of drilling fluid flow rate ${Q}_{\min }$ is 29.6 L/s, namely ${Q}_{r} < {Q}_{\min } < {Q}_{\max }$, which can also be considered as the Case III of the established model in this study. And ${L}_{h\max }$ is 3908 m based on the established model, which is achieved at ${Q}_{\min }$ (Fig. 4a). Therefore, the designed measured depth cannot be drilled if the designed horizontal-section length ${L}_{h0}$ is 4100 m, or even resulting in drilling hazards.

Therefore, the effects of allowable range of drilling fluid flow rate must be considered to establish the more comprehensive and accurate HERL model. Failure to consider the allowable range of drilling fluid flow rate may result in problems of wellbore cleaning or borehole instability, and it is also unclear that what is the main constraint condition for HERL model of horizontal ERW and which kind of HERL model should be adopted.

In this study, the allowable range of drilling fluid flow rate is taken into account to establish a more comprehensive and accurate HERL model of horizontal ERW. Depending on the relationship between the allowable range of drilling fluid flow rate and the rated flow rate of the drilling pump, three kinds of HERLs model are established. Specifically, both the horizontal-section limit based on rated pump pressure ${L}_{h1}$ and the horizontal-section limit based on rated pump power ${L}_{h2}$ should be considered when ${Q}_{\min }\le {Q}_{r}\le {Q}_{\max }$, ${L}_{h1}$ is the main factor when ${Q}_{\min } < {Q}_{\max } < {Q}_{r}$ while ${L}_{h2}$ is the main factor when ${Q}_{r} < {Q}_{\min } < {Q}_{\max }$.

The horizontal-section limit based on rated pump pressure ${L}_{h1}$ first increases and subsequently decreases with the increase in drilling fluid flow rate, whereas the horizontal-section limit based on rated pump power ${L}_{h2}$ keep decreases with the increase in drilling fluid flow rate. In addition, greater rated pump pressure drilling pump *p*_*r*_ means greater ${L}_{h1}$. Similarly, the greater rated pump power *P*_*r*_ corresponds to the greater ${L}_{h2}$. Moreover, both ${L}_{h1}$ and ${L}_{h2}$ show negative correlation with ROP but have positive correlation with drill pipe rotation speed *N*. However, both the ROP and the drill pipe rotation speed have no effects on the rated flow rate of drilling pump *Q*_*r*_. In order to achieve larger HERL, it is necessary to improve the rated pressure of drilling pump *p*_*r*_ and rated power of drilling pump *P*_*r*_ as much as possible; in addition, lower ROP and higher drill pipe rotation speed are also necessary.

For a horizontal ERW, the designed horizontal-section length ${L}_{h0}$ should be less than the maximum horizontal-section limit ${L}_{h\max }$ It is prone to safety hazards if the designed horizontal-section length is longer than the maximum horizontal-section limit which can be achieved in actual drilling operation. Therefore, it is of great significance to accurately predict the HERL by selecting comprehensive and appropriate constraint conditions.

## Method

### Modified model of HERL

#### Constraint conditions

Three constraint conditions for HERL model are given by combining the previous studies and the allowable range of drilling fluid flow rate. They can be also expressed in Eq. (7).

1. When several drilling pumps work together, the actual pump pressure cannot exceed anyone of these rated pump pressures;
2. When several drilling pumps work together, the actual pump power cannot exceed the sum of these rated pump power of all drilling pumps;
3. The drilling fluid flow rate should be within an allowable range of drilling fluid flow rate. On one hand, drilling fluid flow rate should meet the needs of hole cleaning, on the other hand, wellbore pressure should not exceed the bearing capacity of drilled formation.

7 $$\{\begin{array}{c}{p}_{ac}\le \,\min ({p}_{r1},{p}_{r2},\ldots {p}_{rn}),(n=1,2,\ldots )\\ {P}_{ac}\le {P}_{r1}+{P}_{r2}+\ldots +{P}_{rn},\,(n=1,2,\ldots )\\ {Q}_{\min }\le Q\le {Q}_{\max }\end{array}$$

where ${p}_{ac}$ is actual pressure of drilling pump, MPa; *p*_*r*_ is rated pressure of drilling pump, MPa; ${P}_{ac}$ is actual power of drilling pump, kW; ${P}_{r}$ is rated power of drilling pump, kW; $Q$ is drilling fluid flow rate, L/s; ${Q}_{\min }$ is lower limit of drilling fluid flow rate, L/s; ${Q}_{\max }$ is upper limit of drilling fluid flow rate, L/s.

The actual power of drilling pump ${P}_{ac}$ and the rated pressure of drilling pump ${p}_{ac}$ are shown in Eq. (8) and Eq. (9) respectively.

8 $${P}_{ac}={p}_{ac}Q$$

9 $${p}_{ac}={\rm{\Delta }}{p}_{g}+{\rm{\Delta }}{p}_{b}+{\rm{\Delta }}{p}_{st}+{\rm{\Delta }}{p}_{a}$$

where ${\rm{\Delta }}{p}_{g}$ is surface pipeline pressure drop, MPa; ${\rm{\Delta }}{p}_{b}$ is bit pressure drop, MPa; ${\rm{\Delta }}{p}_{st}$ is drill string pressure losses, MPa; ${\rm{\Delta }}{p}_{a}$ is annular pressure losses, MPa. The Eq. (7) can be modified as Eq. (10).

10 $$\{\begin{array}{c}{p}_{ac}={\rm{\Delta }}{p}_{g}+{\rm{\Delta }}{p}_{b}+{\rm{\Delta }}{p}_{st}+{\rm{\Delta }}{p}_{a}\le \,{\rm{\min }}({p}_{r1},{p}_{r2},\ldots {p}_{rn}),(n=1,2,\ldots )\\ {P}_{ac}={p}_{ac}Q\le {P}_{r1}+{P}_{r2}+\ldots +{P}_{rn},(n=1,2,\ldots )\\ {Q}_{\min }\le Q\le {Q}_{\max }\end{array}$$

### HERL based on rated pump pressure

According to the first constraint condition, the actual pump pressure cannot exceed anyone of these rated pump pressures when several drilling pumps work together. The first constraint condition can be expressed in Eqs (11) and (12).

11 $${p}_{ac}={\rm{\Delta }}{p}_{g}+{\rm{\Delta }}{p}_{b}+{\rm{\Delta }}{p}_{st}+{\rm{\Delta }}{p}_{a}\le \,{\rm{\min }}({p}_{r1},{p}_{r2},\ldots {p}_{rn}),(n=1,2,\ldots )$$

12 $${p}_{ac}={\rm{\Delta }}{p}_{g}+{\rm{\Delta }}{p}_{b}+\sum _{i=1}^{j}\frac{{\rm{\Delta }}{p}_{sti}}{{\rm{\Delta }}{L}_{i}}{L}_{i}+\sum _{i=1}^{j}\frac{{\rm{\Delta }}{p}_{ai}}{{\rm{\Delta }}{L}_{i}}{L}_{i}\le \,{\rm{\min }}({p}_{r1},{p}_{r2},\ldots {p}_{rn}),(n=1,2,\ldots )$$

where $\frac{{\rm{\Delta }}{p}_{sti}}{{\rm{\Delta }}{L}_{i}}$ is drill string pressure loss gradients in several parts of drill string, MPa/m; $\frac{{\rm{\Delta }}{p}_{ai}}{{\rm{\Delta }}{L}_{i}}$ is annular pressure loss gradients in several parts of annulus, MPa/m.

For a horizontal ERW, we mainly analyze its horizontal-section limit ${L}_{h}$. If merely one drilling pump is considered, its rated pressure is *p*_*r*_, the horizontal-section limit based on rated pump pressure ${L}_{h1}$ under the first constraint condition can be expressed in Eq. (13).

13 $${L}_{h1}=\frac{{p}_{r}-{\rm{\Delta }}{p}_{g}-{\rm{\Delta }}{p}_{b}-({\rm{\Delta }}{p}_{stv}+{\rm{\Delta }}{p}_{std})-({\rm{\Delta }}{p}_{av}+{\rm{\Delta }}{p}_{ads}+{\rm{\Delta }}{p}_{adl})}{{(\frac{{\rm{\Delta }}p}{{\rm{\Delta }}L})}_{sth}+{(\frac{{\rm{\Delta }}p}{{\rm{\Delta }}L})}_{ah}}$$

where ${\rm{\Delta }}{p}_{stv}$ is drill string pressure losses of vertical section, MPa; ${\rm{\Delta }}{p}_{std}$ is drill string pressure losses of deviated sections, MPa; ${\rm{\Delta }}{p}_{av}$ is annular pressure losses of vertical section, MPa; ${\rm{\Delta }}{p}_{ads}$ is annular pressure losses of small-inclination section, MPa; ${\rm{\Delta }}{p}_{adl}$ is annular pressure losses of large-inclination section, MPa; ${({\rm{\Delta }}p/{\rm{\Delta }}L)}_{sth}$ is drill string pressure loss gradients in horizontal section, MPa/m; ${({\rm{\Delta }}p/{\rm{\Delta }}L)}_{ah}$ is annular pressure loss gradients in horizontal section, MPa/m. Their calculation methods refer to the following literatures (Wang and Liu, 1995; Kelessidis *et al*., 2011; Fan, 2013; Erge *et al*., 2015)^8–11^.

### HERL based on rated pump power

According to the second constraint condition, the actual pump power cannot exceed the sum of these rated pump power of all drilling pumps when several drilling pumps work together. The second constraint condition can be expressed in Eqs (14) and (15).

14 $${P}_{ac}={p}_{ac}Q\le {P}_{r1}+{P}_{r2}+\ldots +{P}_{rn},(n=1,2,\ldots )$$

15 $${P}_{ac}={p}_{ac}Q=({\rm{\Delta }}{p}_{g}+{\rm{\Delta }}{p}_{b}+\sum _{i=1}^{j}\frac{{\rm{\Delta }}{p}_{sti}}{{\rm{\Delta }}{L}_{i}}{L}_{i}+\sum _{i=1}^{j}\frac{{\rm{\Delta }}{p}_{ai}}{{\rm{\Delta }}{L}_{i}}{L}_{i})Q\le {P}_{r1}+{P}_{r2}+\ldots +{P}_{rn},(n=1,2,\ldots )$$

For a horizontal ERW, we mainly analyze its horizontal-section limit ${L}_{h}$. Similarly, if merely one drilling pump is considered, its rated power is *P*_*r*_, the horizontal-section limit based on rated pump power ${L}_{h2}$ under the second constraint condition can be expressed in Eq. (16).

16 $${L}_{h2}=\frac{\frac{{P}_{r}}{Q}-{\rm{\Delta }}{p}_{g}-{\rm{\Delta }}{p}_{b}-({\rm{\Delta }}{p}_{stv}+{\rm{\Delta }}{p}_{std})-({\rm{\Delta }}{p}_{av}+{\rm{\Delta }}{p}_{ads}+{\rm{\Delta }}{p}_{adl})}{{(\frac{{\rm{\Delta }}p}{{\rm{\Delta }}L})}_{sth}+{(\frac{{\rm{\Delta }}p}{{\rm{\Delta }}L})}_{ah}}$$

### Allowable range of drilling fluid flow rate

According to the third constraint condition, the actual drilling fluid flow rate should be within an allowable range of drilling fluid flow rate. On one hand, the drilling fluid flow rate should meet the needs of hole cleaning. On the other hand, the wellbore pressure should not exceed the bearing capacity of drilled formation.

#### Upper limit

In general, greater drilling fluid flow rate often means better state of hole cleaning. However, there is an upper limit for drilling fluid flow rate since the too high drilling fluid flow rate poses a great threat to the bearing capacity of drilled formation due to the exceeded annular drilling fluid velocity. The upper limit of drilling fluid flow rate ${Q}_{\max }$, namely the upper limit considering the bearing capacity of drilled formation can be determined based on the open hole extended-reach limit (OHERL) theory.

The OHERL theory can be summarized as follows. The horizontal ERW cannot extend without limitation, the drilled formation will be fractured if the bottom hole pressure exceeds the fracture pressure, which is a critical point, and it can be expressed in Eq. (17)^7, 12–14^.

17 $$0.00981[{\rho }_{s}{C}_{s}+{\rho }_{m}(1-{C}_{s})]{D}_{v}+({\rm{\Delta }}{p}_{av}+\sum _{i=1}^{j}{\rm{\Delta }}{p}_{adi}+{\rm{\Delta }}{p}_{ah})=0.00981{\rho }_{f}{D}_{v}$$

where ${\rho }_{s}$ is solids density, namely cuttings density, g/cm3; ${\rho }_{m}$ is drilling fluid density, g/cm3; ${C}_{s}$ is solid volumetric concentration, %; ${\rm{\Delta }}{p}_{av}$ is annular pressure losses of vertical section, MPa; ${\rm{\Delta }}{p}_{adi}$ are annular pressure losses of several deviated sections, MPa; ${\rm{\Delta }}{p}_{ah}$ annular pressure losses of horizontal section at the critical point, MPa.

In general, the horizontal-section limit based on OHERL theory should be larger than the designed horizontal-section length ${L}_{h0}$. The limit values of drilling fluid flow rate can be obtained when the horizontal-section limit based on OHERL theory equals ${L}_{h0}$, which is obtained from Eq. (18).

18 $$\frac{0.00981{\rho }_{f}{D}_{v}-0.00981[{\rho }_{s}{C}_{s}+{\rho }_{m}(1-{C}_{s})]{D}_{v}-({\rm{\Delta }}{p}_{av}+\sum _{i=1}^{j}{\rm{\Delta }}{p}_{adi})}{{({\rm{\Delta }}p/{\rm{\Delta }}L)}_{ah}}={L}_{h0}$$

The results show that there are two limit values of drilling fluid flow rate, the larger of which can be taken as the upper limit of drilling fluid flow rate ${Q}_{\max }$, namely the upper limit considering the bearing capacity of drilled formation.

#### Lower limit

If the drilling fluid flow rate is too small, the hole cleaning condition becomes worse. Moreover, both the annular pressure losses and the bottom hole pressure are increased, which will also pose a great threat to the drilled formation. Therefore, two factors should be considered to determine the lower limit of drilling fluid flow rate. On one hand, the lower limit considering the bearing capacity of drilled formation ${Q}_{\min -df}$ can be obtained based on the above OHERL theory. On the other hand, considering the needs of hole cleaning, the lower limit based on the needs of hole cleaning ${Q}_{hc}$ can be obtained. The lower limit of drilling fluid flow rate ${Q}_{\min }$ can be obtained by Eq. (19).

19 $${Q}_{\min }=\,{\rm{\max }}({Q}_{\min -df},{Q}_{hc})$$

The lower limit considering the bearing capacity of drilled formation ${Q}_{\min -df}$ can also be determined by the OHERL theory. The lower limit value of drilling fluid flow rate which satisfies the Eq. (18) can be regarded as the ${Q}_{\min -df}$.

The horizontal ERW requires a certain amount of annular drilling fluid flow rate in order to meet the needs of hole cleaning, so there exists a lower limit based on the needs of hole cleaning ${Q}_{hc}$. The predecessors have made lots of researches in this field (Li and Liu, 1994; Larsen *et al*., 1997; Wang and Song, 2003; Li *et al*., 2010)^15–18^. According to the difference of deviation angle and annular size, the authors divide the whole sections of horizontal ERW into the vertical and small inclination section, the large inclination section, and the horizontal-section (Xin *et al*., 2016c)^13^. Therefore, ${Q}_{\min 1}$ (the lower limit of drilling fluid flow rate in vertical section and small-inclination section), ${Q}_{\min 2}$ (the lower limit of drilling fluid flow rate in large-inclination section) and ${Q}_{\min 3}$ (the lower limit of drilling fluid flow rate in horizontal section) can be achieved. The minimum one can be taken as the lower limit of drilling fluid flow rate ${Q}_{\min }$, which is given by Eq. (20).

20 $${Q}_{hc}=\,{\rm{\max }}({Q}_{{\rm{min1}}},{Q}_{{\rm{min2}}},{Q}_{{\rm{min3}}})$$

**Figure 5:**
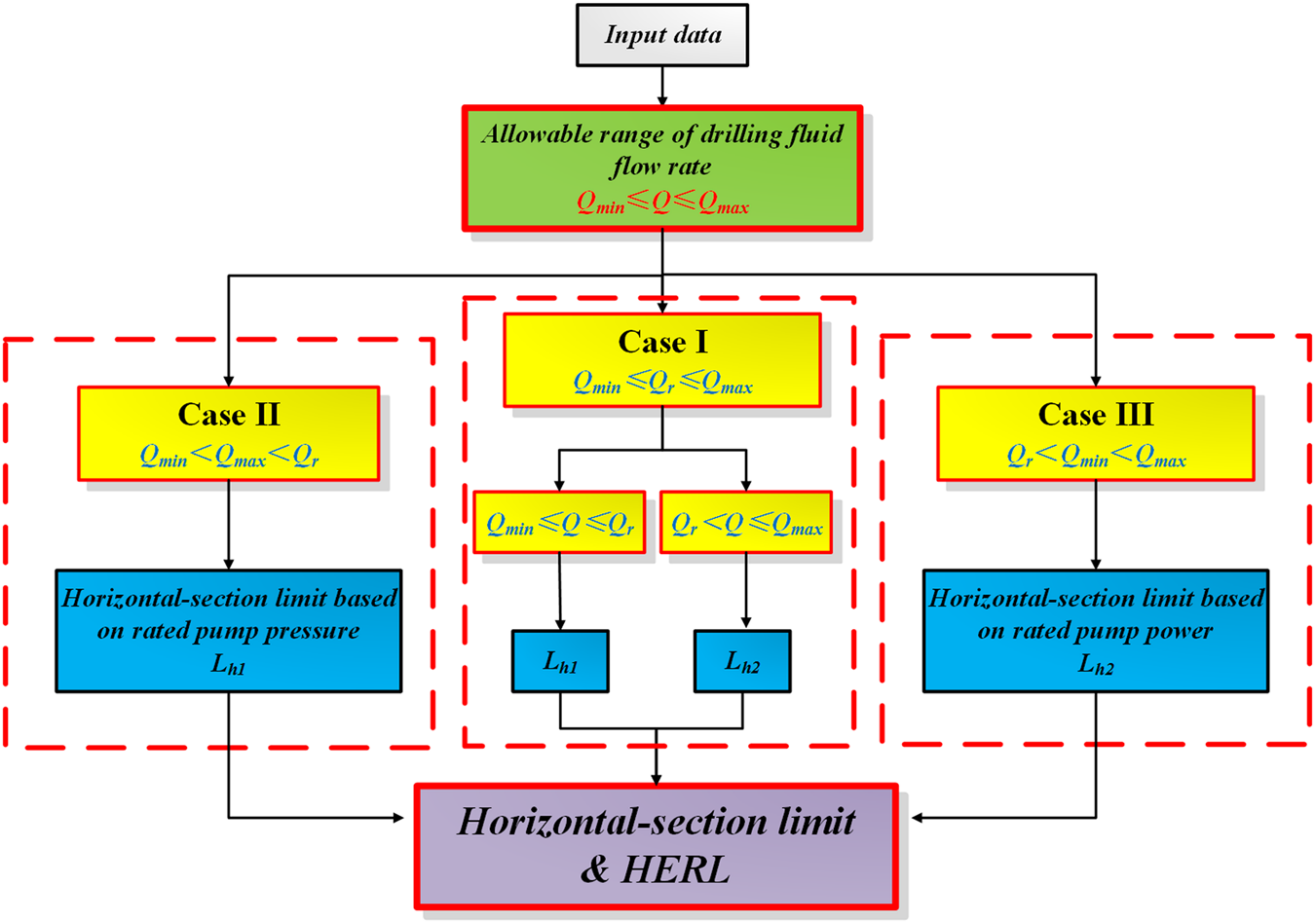
Flow chart of the calculation procedure.

The calculation procedure is summarized as follows and shown in Fig. 5.

1. Determine the allowable range of drilling fluid rate ${Q}_{\min }\le Q\le {Q}_{\max }$ by considering the needs of hole cleaning and the bearing ability of drilled formation;
2. Determine the relationship between ${Q}_{\min }\le Q\le {Q}_{\max }$ and the rated flow rate of the drilling pump *Q*_*r*_;
3. Determine which situation the HERL belongs to and which kind of HERL model is to be adopted, then calculate the horizontal-section limit ${L}_{h}$ of the horizontal ERW;
4. If ${Q}_{\min }\le {Q}_{r}\le {Q}_{\max }$ (Case I), calculate ${L}_{h}$ using Eq. (4); if ${Q}_{\min } < {Q}_{\max } < {Q}_{r}$ (Case II), calculate ${L}_{h}$ using Eq. (5); if ${Q}_{r} < {Q}_{\min } < {Q}_{\max }$ (Case III), calculate ${L}_{h}$ using Eq. (6).

## References

[1] Martins, A. L. *et al*. Hydraulic Limits for Drilling and Completing Long Horizontal Deepwater Wells. In *SPE International Thermal Operations and Heavy Oil Symposium and Western Regional Meeting*. Society of Petroleum Engineers (2004, January).

[2] Wang Z, Guo X (2008). Study on hydraulic extended-reach limit of ERW. Drilling & Production Technology..

[3] Guo X, Wang Z (2009). The hydraulic extended limit of mega-extended-reach well at Liuhua Field in South China Sea. Oil Drilling & Production Technology..

[4] Gao D, Tan C, Tang H (2009). Limit analysis of extended reach drilling in South China Sea. Petroleum Science..

[5] Sun, T. Research on Well Trajectory Design & Control in Horizontal Drilling. Doctoral dissertation in China University of Petroleum, Beijing (2013).

[6] Chen, T. & Guan, Z. Theory and technology of drilling engineering. *The Press of the University of Petroleum*, *Dongying* (*China*). 155–157 (2000).

[7] Li X, Gao D, Zhou Y, Cao W (2016). General approach for the calculation and optimal control of the extended-reach limit in horizontal drilling based on the mud weight window. Journal of Natural Gas Science and Engineering..

[8] Wang H, Liu X (1995). Analysis of annular pressure losses in horizontal section. West-China Exploration Engineering..

[9] Kelessidis VC, Dalamarinis P, Maglione R (2011). Experimental study and predictions of pressure losses of fluids modeled as Herschel–Bulkley in concentric and eccentric annuli in laminar, transitional and turbulent flows. Journal of Petroleum Science and Engineering..

[10] Fan, H. *Practical Drilling Fluid Mechanics*. Petroleum Industry Press, Beijing (2013).

[11] Erge, O. *et al*. The Effects of Drillstring-Eccentricity,-Rotation, and-Buckling Configurations on Annular Frictional Pressure Losses While Circulating Yield-Power-Law Fluids. *SPE Drilling & Completion* (2015).

[12] Li X, Gao D, Zhou Y, Zhang H (2016). A model for extended-reach limit analysis in offshore horizontal drilling based on formation fracture pressure. Journal of Petroleum Science and Engineering..

[13] Li X, Gao D, Zhou Y, Zhang H (2016). Study on open-hole extended-reach limit model analysis for horizontal drilling in shales. Journal of Natural Gas Science and Engineering..

[14] Li X, Gao D, Zhou Y, Zhang H, Yang Y (2017). Study on the prediction model of the open-hole extended-reach limit in horizontal drilling considering the effects of cuttings. Journal of Natural Gas Science and Engineering..

[15] Li H, Liu X (1994). The determination method for reasonable return velocity of horizontal drilling. Journal of China University of Petroleum (Edition of Natural Sciences)..

[16] Larsen TI, Pilehvari AA, Azar JJ (1997). Development of a new cuttings-transport model for high-angle wellbores including horizontal wells. SPE Drilling & Completion..

[17] Wang D, Song X (2003). The model of hole cleaning in inclined well. Petroleum Drilling Techniques..

[18] Li Q, Li Q, Wang L, Zhang B (2010). Research on the model and application of hole cleaning in inclined well. Journal of Xi’an Shiyou University (Natural Science Edition)..

